# The use of non‐vitamin K oral anticoagulants in dialysis patients—A systematic review

**DOI:** 10.1111/sdi.13098

**Published:** 2022-05-27

**Authors:** Agitha Chandrasegaram, Christian Daugaard Peters

**Affiliations:** ^1^ Department of Renal Medicine Aarhus University Hospital Aarhus; ^2^ Department of Clinical Medicine Aarhus University Aarhus

## Abstract

Non‐vitamin K oral anticoagulants (NOACs) are used for prevention of thromboembolic events, but their use in dialysis patients is debatable. This study investigated the available evidence for the use of NOACs in dialysis patients. Online databases were systematically searched for eligible studies including pharmacokinetic (PK) studies, cohort studies, and randomized control trials (RCTs) comparing NOAC with vitamin K antagonist (VKA) or no anticoagulant treatment. Newcastle Ottawa Scale and Cochrane Risk of bias tool were used for quality assessment. Twenty studies were identified (nine PK studies, two RCTs, and nine cohort studies). Most of the studies investigated apixaban or rivaroxaban. In dialysis patients, less accumulation was reported with apixaban and rivaroxaban compared to dabigatran and edoxaban. PK studies indicate that high dose apixaban or rivaroxaban should be avoided. The two RCTs (rivaroxaban/apixaban vs. VKA) were small and underpowered regarding stroke and bleeding outcomes. Most cohort studies found apixaban superior to VKA, whereas comparison of rivaroxaban with VKA yielded conflicting results. Cohort studies comparing apixaban high dose (5 mg) with low dose (2.5 mg) twice daily suggest a lower risk of stroke with high dose but also a higher risk of bleeding with high dose. Apixaban versus no anticoagulation was compared in one cohort study and did not lower the risk of stroke compared with non‐treated regardless of apixaban dosage. Widespread use of NOACs in dialysis patients is limited by adequately sized RCTs. Available evidence suggests a potential for use of apixaban and rivaroxaban in reduced dose.

## 1 INTRODUCTION

**FIGURE 1:**
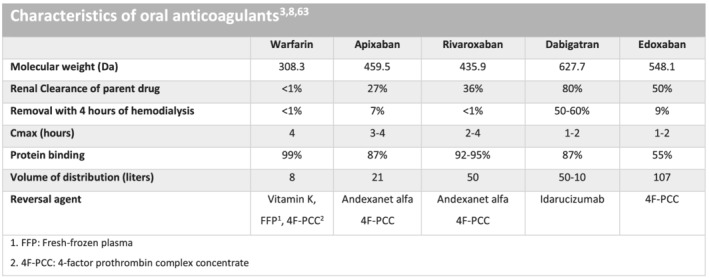
Characteristics of oral anticoagulants.^3^, ^8^, ^10^ ^1^FPP: fresh‐frozen plasma. ^2^4F‐PCC: four‐factor prothrombin complex concentrate

Patients with chronic kidney disease (CKD) on dialysis treatment have a high prevalence of atrial fibrillation (AF).^1^ Despite the high prevalence of AF, it is unclear whether dialysis patients should be anticoagulated. Non‐vitamin K anticoagulants (NOACs) also known as direct oral anticoagulants (DOACs) are often used to prevent stroke in patients with AF^2^ and as prophylaxis after thromboembolic events such as deep vein thrombosis and lung embolism.^3^ Four different NOACs, dabigatran,^4^ rivaroxaban,^5^ apixaban,^6^ and edoxaban^7^ are currently available. Dabigatran is an oral direct thrombin inhibitor, whereas rivaroxaban, edoxaban, and apixaban are direct factor Xa inhibitors.^4^, ^5^, ^6^, ^7^ In non‐renal patients, NOACs are now mainly used instead of a vitamin K antagonist (VKA) such as warfarin.^8^ NOACs are easier to handle, since regular blood sampling for monitoring is not necessary unlike VKAs where the narrow therapeutic range is affected by several food and drug interactions.^9^ All NOACs are in varying degrees renally excreted; however, pharmacokinetic (PK) properties are somewhat different in terms of renal clearance, the degree of protein binding, and the potential for removal by dialysis treatment as outlined in Figure 1.^3^, ^8^

Accordingly, there is a potential for drug accumulation if NOACs are used in patients with acute or chronic renal failure.^11^ The effect and safety of NOACs compared with VKA treatment with warfarin was investigated in four large phase 3 trials in patients with AF: RE‐LY,^12^ ROCKET‐AF,^13^ ARISTOTLE,^14^ and ENGAGE AF‐TIMI 48.^15^ However, none of these included end‐stage renal disease (ESRD) patients on dialysis treatment. Currently, the European Heart Rhythm Association does not recommend use of NOACs in CKD patients with creatinine clearance (CrCl) ≤ 15 ml/min^16^ and dialysis patients with AF are therefore in Europe predominantly treated with VKA. Yet, a large proportion of dialysis patients with AF receive no anticoagulation, due to the lack of available evidence and the known risks with VKA treatment.^16^, ^17^, ^18^

Both NOAC and VKA treatment increase the bleeding risk significantly, which is a major hazard to the patients regardless of the drug used, and most cohort studies in dialysis patients have failed to demonstrate a benefit from VKA treatment in terms of stroke prevention.^19^, ^20^, ^21^, ^22^, ^23^ Besides, VKA treatment, unlike NOACs, increases the risk of calcifylaxis, a life‐threatening syndrome of vascular calcification, typically attributed to warfarin‐induced deficiency of vitamin K‐dependent calcification inhibitors.^24^

Even without anticoagulant therapy, ESRD patients on dialysis have increased risk of bleeding usually attributed to uremia‐induced platelet dysfunction and impaired interaction between platelets and the vessel wall.^25^ Adding to this are factors such as frailty, comorbidity, high prevalence of antiplatelet drugs,^26^, ^27^, ^28^ and the regular use of low‐molecular‐weight heparin (LMWH) given in order to minimize clotting in the dialysis filter during hemodialysis (HD) treatment.^29^ Thus, when comparing bleeding risk in dialysis patients, HD patients have a 1.5‐fold higher risk compared with peritoneal dialysis (PD) patients which could be attributed to LMWH.^30^ HD patients also have a higher underlying risk of bleeding due to intermittent puncture of the vascular access with needles.^30^ A previous bleeding history is another important risk factor, and dialysis patients belonging to this subgroup are reported to be at the highest risk of a new bleeding event.^30^, ^31^, ^32^

Consequently, the added benefit of stroke prevention in dialysis patients with AF could be outbalanced by an increased risk of bleeding.^9^ This, in conjunction with absence of large‐scale randomized controlled trials (RCTs) in ESRD, makes use of NOACs in dialysis patients debatable. This debate has recently intensified after the Food and Drug Administration (FDA) approved use of apixaban in ESRD^33^ causing increased utilization of apixaban among dialysis patients in the United States.^34^

The aim of this systematic review was to search the existing literature in order to find out whether NOACs can be safely used in dialysis patients and if the PKs of NOACs are significantly different in dialysis patients. An additional aim was to locate intervention studies in dialysis patients comparing NOAC with VKA treatment including upcoming studies.

## 2 MATERIALS AND METHODS

### 2.1 Search strategy and data extraction

Published articles were searched from December 2020 to December 2021 in the following databases: PubMed, Cochrane, Embase, and Web of Science. The ClinicalTrials.gov database was used to find ongoing or unpublished studies. PRISMA 2020 checklist was used in the search for eligible studies using different combinations of MeSH terms and keywords (See supplements). Beforehand, we decided to include PK studies, RCTs, and observational cohort studies without any restrictions regarding year of publication. Case reports, review articles, editorials, and guidelines were excluded alongside papers not written in English. References of review articles and studies were manually searched for additional studies. Two studies were identified via the list of references. One upcoming Swedish RCT (SACK)^35^ was identified by chance via e‐mail correspondence with colleagues which led us to the Svensk Njurmedicinsk Förening (Swedish Renal Medicine Association) webpage for study details. One author screened the searched results according to eligibility and independently reviewed titles and abstracts. The full‐text review was conducted on studies that met the eligibility criteria by both authors. For stroke outcomes, the type and incidence/ rate/ risk of stroke were extracted. For bleeding outcomes, data about the severity, definition, and incidence/ rate/ risk of bleeding or mortality were extracted. Any discrepancies were resolved by discussion and consensus. According to Danish law, approval from an ethics committee and informed consent from the patients were not required for this study.

### 2.2 Study selection

**FIGURE 2:**
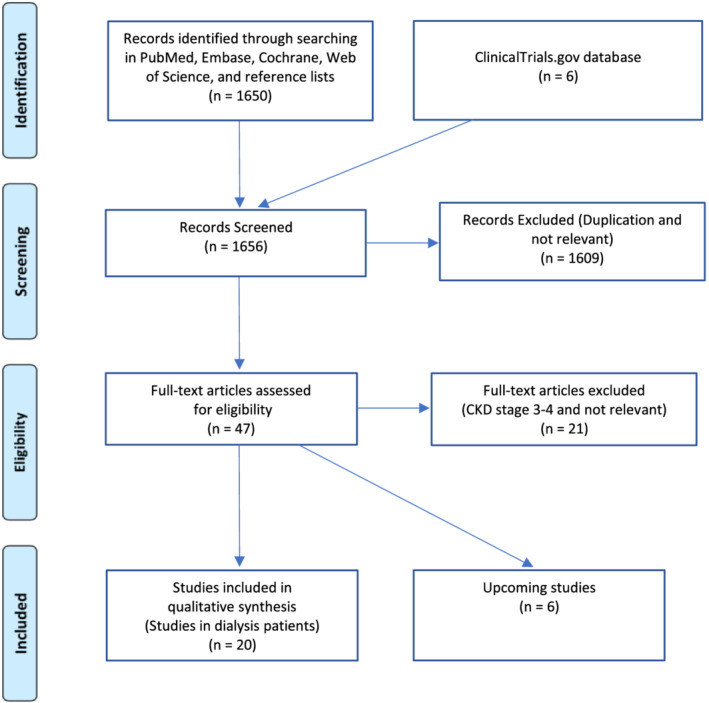
Selection of studies based on PRISMA‐guidelines

We screened a total number of 1650 articles (Figure 2). NO PK studies in PD patients were found except for the ApiDP trial registered at the ClinicalTrials.gov database but without published data and could therefore not be included. An open‐label RCT conducted in HD patients (RENAL‐AF) was also found via ClinicalTrials.gov. Results from this study have not been published yet but were presented at the American Heart Association Annual meeting in 2019 (AHA 2019). By searching the title name in Pubmed, an abstract with PK results was found and a presentation with the trial results was found via the American College of Cardiology website. Two upcoming studies about apixaban versus warfarin in dialysis patients and two with warfarin versus no anticoagulant treatment were identified via ClinicalTrials.gov. After screening and removal of duplicates, 47 studies (combination of PKs studies, RCTs, and observational cohort studies and upcoming studies) were at first hand eligible for inclusion. Finally, after excluding 21 studies conducted in CKD stage 3–4 patients, only 20 studies were found eligible, and six studies were ongoing/recruiting without results (five RCTs and one PK study).

### 2.3 Risk of bias in the studies

Newcastle Ottawa Scale (NOS) was used to assess the risk of bias, and thereby, the quality of all eight cohort studies was included.^36^ NOS ranks the studies with a star system based on three categories. The categories are (1) selection of study groups, (2) the comparability of the groups, and (3) the ascertainment of either outcome or exposure of interest. A high number of stars awarded to one study indicate that the study has high quality. The two RCTs included were quality assessed using the Cochrane Risk of Bias tool (ROSB).^37^ The tool is based on seven categories: random sequence generation, allocation concealment, selective reporting, other sources of bias, blinding of participants and personnel, blinding of outcome assessment, and incomplete outcome data. Each category is evaluated as having high, low, or unclear risk of bias.

## 3 RESULTS

### 3.1 Study characteristics

An overview of all selected studies such as study type (PKs study, retrospective cohort study, or RCT), number of patients, dialysis modality, and outcomes (bleeding and stroke) are provided in Tables 1, 2, 3. Upcoming studies are provided in Table 4.

**TABLE 1:** Characteristics of nine pharmacokinetic studies in dialysis patients included in this review

NOAC	Study	Study design	Study subjects	Oral dose	Treatment	Outcomes
Apixaban	Wang et al.^38^ 2016	Open‐label	8 ESRD on HD 8 normal renal function	5 mg dose once daily to healthy subjects 5 mg BID to ESRD subjects.	On dialysis: Day 1 period 1 2 h predialysis 5 mg. Off‐dialysis: Day 1 period 2 5 mg immediately after 4 h dialysis.	On dialysis: Mean $\mathrm{AU}C_{\infty }$ 14% and Cmax 13% in ESRD group. Off dialysis: Mean $\;\mathrm{AU}C_{\infty }$ 36% higher, Cmax 10% lower in ESRD group. Mean T_1/2_ ca. 13 h in ESRD group and 20 h in healthy subjects. Dialysate clearance 17.7 mL/min in ESRD group and renal clearance 11.3 mL/min healthy group. 6.7% of the apixaban dose was in the dialysate.
Apixaban	Mavrakanas et al.^39^ 2017	Open‐label	7 with ESRD on HD	2.5 mg or 5 mg BID	Phase 1 (non‐dialysis): 7 patients ‐ 2,5 mg twice daily for 8 days. Phase 2: On day 9, 6 of 7 patients ‐ 2,5 mg immediately before dialysis. Phase 3: 5 patients from phase 2 after a 5‐day washout period, received 5 mg twice daily for 8 days.	Phase 1: ${\mathrm{AUC}}_{\infty -24}\;$ increased: 629 to 2054 ng h/ml. C_through_ 45 to 132 ng/ml. Phase 2: 4% of apixaban was removed (comparison of mean drug levels during day 8 and day 9). Phase 3: ${\mathrm{AUC}}_{\infty -24}$ increased to 6,054 ng h/ml compared with steady‐state dose. Accumulation of 2.5 mg apixaban was similar to 5 mg apixaban in healthy subjects in ARISTOLE study. 5 mg twice daily led to supratherapeutic levels.
Apixaban	Van den Bosch et al.^40^ 2020	Open‐label	24 ESRD on HD	2.5 mg or 5 mg BID	30 min before/immediately after dialysis on the midweek dialysis days. (8 in each group).	Apixaban 5 mg pre dialysis resulted in higher AUC in comparison with 2.5 mg. Mean ${\mathrm{AUC}}_{\infty -48}$ predialysis was 48% (2.5 mg) and 26% (5 mg) lower than postdialysis. 2.5 mg postdialysis and 5 mg predialysis similar ${\mathrm{AUC}}_{0-48}\;$ 2.5 mg predialysis and 5 mg postdialysis significant different ${\mathrm{AUC}}_{\infty -48}$ C_max_ was 32% higher in postdialysis versus predialysis 2.5 mg, and 19% higher in 5 mg treated post versus predialysis group.
Apixaban	Pokorney et al.^41^ 2020	RCT	63 ESRD on HD	2.5 mg or 5 mg BID	2.5 mg to subjects ≥80 years or weight ≤ 60 kg otherwise 5 mg.	Mean AUC 5452 ng h/ml (5 mg dose). Mean AUC 2990 ng h/ml (2.5 mg dose). Modestly higher apixaban levels on high dose (5 mg) but consistent with ARISTOLE study.
Rivaroxaban	De Vriese et al.^42^ 2015	Cohort study	18 ESRD on HD	Multiple versus single dose 10 mg	1) 10 mg after 3 dialysis session. 2) 10 mg 6–8 h before dialysis session. 3) From 1 to 7 days 10 mg daily. AUC measured during 24 h each day. Dialysis on day 2, 4 and 6.	1) Mean ${\mathrm{AUC}}_{0-44}$ 2.072 𝜇g/L/h, mean C_max_ 172,6 𝜇g/L and mean T_1/2_ 8.6 h. 2) Dialysate Mean concentration 5.4 ± 5.4 (SD) ng/ml. 3) mean ${\mathrm{AUC}}_{0-24}\;$ day 1: 1.499 𝜇g/L/h, mean C_max_ 152.8 𝜇g/L and mean T_1/2_ 6.6 h. Mean ${\mathrm{AUC}}_{0-24}$ day 7: 1.851 𝜇g/L/h, mean C_max_ 192.0 𝜇g/L and mean T_1/2_ 7.3 h. Multiple dose: Mean C_thorugh_ 20.2 mg/L. Rivaroxaban dose of 10 mg in HD patients without residual kidney function results in drug exposure similar to 20 mg in healthy volunteers. Rivaroxaban was not eliminated by dialysis.
Rivaroxaban	Dias et al.^43^ 2016	Open‐label	Total: 16 Group A: 8 ESRD on HD Group B: 8 healthy matched	15 mg	Group A: Period 1: 15 mg 2 ± 0.5 h predialysis. Period 2: 15 mg 3 h postdialysis. Group B: Period 1: Rivaroxaban with predose and postdose PK samples taken.	AUC increased 56% postdialysis in group A versus B. approx. 35% decrease in drug clearance in ESRD subjects. AUC 5% lower for predialysis versus postdialysis. Renal function had a little impact on C_max._ The ratio of active transport to passive filtration of unchanged rivaroxaban was 3:1.
Dabigatran	Khadzhynov et al.^44^ 2013	Open‐label	7 (ESRD and without AF)	Multiple dose dabigatran etexilate 150 mg 110 mg 75 mg	On day 1: 150 mg immediately after hemodialysis (0 h start). On day 2: 110 mg (after 21 h). On day 3: 75 mg (after 42 h) 4 h dialysis on day 1 before the dose, and day 3, and 5. On day 3 – different target blood flow rate of 200 mL/min (period 1) and 400 mL/min (period 2).	4 h hemodialysis with 200 mL/min flow rate and 350–395 mL/min flow rate cleared 48,8% and 59,3% fraction of dabigatran from plasma. The anticoagulant activity reduced proportionally to the HD related reduction in plasma levels. 7.5–15.5% increase in dabigatran plasma concentration after dialysis.
Dabigatran	Stangier et al.^45^ 2010	Open‐label	6 ESRD on HD 23 renal impairment 6 healthy	dabigatran etexilate 50 mg (ESRD) 150 mg (renal impairment)	ESRD group: 50 mg before the start of 4‐hour dialysis session. Renal impairment: 150 mg to renal impairment, and healthy subjects.	C_max_ was low (13.5 ng/ml) and ${\mathrm{AUC}}_{\infty }$ was 2‐fold greater, and T_½_ was more than 2‐fold longer in ESRD on dialysis compared to healthy subjects. ${\mathrm{AUC}}_{0-\infty }$ was 6‐fold greater in severe renal impairment patients. Mean fraction of dabigatran in dialysis fluid: 0,44%. 1,9% in healthy subjects. Mean fraction of drug removed from blood by dialysis was 62% ‐68%.
Edoxaban	Parasrampuria et al.^46^ 2015	Open‐label	10 ESRD on HD	15 mg	Two occasions: On dialysis: Intake 2 h before a 4 h HD. Off‐dialysis: Intake between the dialysis session. One group: On dialysis dose, ≥7 days washout, off‐dialysis dose. Second group: Off‐dialysis dose, ≥7 days washout, on dialysis dose.	Mean $\mathrm{AU}C_{0-\infty }\;$ 676.2 ng h/ml, mean C_max_ 53,3 ng/ml, and mean T_½_ 10.6 h (on dialysis). Mean $\mathrm{AU}C_{0-\infty }$ 691,7 ng h/ml, mean C_max_ 56,3 ng/ml, and mean T_½_ 10.4 h (off dialysis). Dialyser clearance 5.7 L/h and HD clearance was 6.1 L/h.

Abbreviations: AUC, area under the curve; BID, (bis in die) = twice a day; ESRD, end‐stage renal disease; HD, hemodialysis; NOAC, non‐vitamin K oral anticoagulant.

**TABLE 2:** Stroke outcomes for NOAC use in dialysis patients and characteristics of 10 studies included in the review

NOAC	Study and year of publication	Study design	Mean follow‐up (years)	Treatment group (number of patients)	Renal function	Oral dose	Comparison group (number of patients)	Definition of stroke	Incidence: Treatment group and comparison group	HR of treatment group versus comparison group (95% cl)
Apixaban	Pokorney et al.^47^ 2019 (RENAL‐AF)	RCT	1	Apixaban: 82 5 mg BID: 55 2.5 mg BID: 22 Apixaban reduced from 5.0–2.5 mg BID: 15	ESRD on HD	5 mg or 2.5 mg BID	Warfarin: 72	Ischemic or hemorrhagic stroke	Apixaban: 2.4% Warfarin: 2.8%	Not reported
Apixaban	Stanton et al.^48^ 2017	Retrospective cohort	Apixaban: 1.01 Warfarin: 1.54	Apixaban: 73 5 mg BID or 10 mg BID: 27 2.5 mg BID: 45	HD/PD/CrCL<25 mL/min or SCr of >2.5 mg/dL	10 mg or 5 mg or 2.5 mg BID	Warfarin: 73	Ischemic stroke	Apixaban: 7.5% Warfarin: 7.5%	No difference
Apixaban	Reed et al.^49^ 2018	Retrospective cohort	0.83	Apixaban: 74 5 mg BID: 59 2.5 mg BID: 15	ESRD on HD/PD)	5 mg or 2.5 mg BID	Warfarin: 50	Ischemic stroke	Apixaban: 0% Warfarin: 0%	Not reported
Apixaban	Siontis et al.^34^ 2018	Retrospective cohort	Apixaban: 0.29 Warfarin: 0.43	Apixaban: 2351 5 mg BID: 1034 2.5 mg BID: 1317	ESRD on HD and PD	5 mg or 2.5 mg BID	Warfarin: 7053	Ischemic stroke or systemic embolism	Apixaban 12.4/100 patient‐years Warfarin: 11.8/100 patients‐years Apixaban 5 mg BID versus warfarin Apixaban 8.8/100 patient‐years Warfarin: 11.8/100 patient‐years Apixaban 2.5 mg BID versus warfarin Apixaban 15.3/100 patient‐years Warfarin: 11.8/100 patient‐years	Apixaban versus warfarin 0.88 (0.69–1.12) Apixaban 5 mg BID versus warfarin 0.64 (0.42–0.97) Apixaban 2.5 mg BID versus warfarin 1.11 (0.87–1.33) Apixaban: 5 mg BID versus 2.5 mg BID 0.61 (0.37–0.98)
Apixaban	Mavrakanas et al.^50^ 2020	Retrospective cohort	0.31	Apixaban: 521 5 mg BID: 207 2.5 mg BID: 257	PD or HD	5 mg or 2.5 mg BID	No anticoagulation treatment: 1561	Ischemic or hemorrhagic stroke, transient ischemic attack or systemic tromboemolism	Apixaban versus no anticoagulation treatment: Apixaban 7.5/100 patient‐years No anticoagulation 7.0/100 patient‐years Apixaban 5 mg versus no treatment: Apixaban 3.4/100 patient‐years No anticoagulation 4.8/100 patient‐years. Apixaban 2.5 mg versusno treatment: Apixaban 4.5/100 patient‐years No anticoagulation 4.7/100 patient‐years	Apixaban versus no anticoagulation treatment: 1.24 (0.69–2.23) Apixaban 5 mg versus no treatment: 0.90 (0.20–4.01) Apixaban 2.5 mg versus no treatment: 1.11 (0.39–3.17)
Rivaroxaban and apixaban	Miao et al.^51^ 2020	Retrospective cohort	0.87	Rivaroxaban: 787 Apixaban: 1836	ESRD on HD	Rivaroxaban: < 20 mg/day Apixaban: <10 mg/day		Ischemic or hemorrhagic stroke	Rivaroxaban: 1.27/100 person‐years Apixaban: 1.26/100 person‐years	1.12 (0.45–2.76)
Rivaroxaban and dabigatran	Chan et al.^9^ 2015	Retrospective cohort	Dabigatran: 0.44 Rivaroxaban: 0.30Warfarin: 0.48	Rivaroxaban: 244 Dabigatran: 281	ESRD on HD	20 mg/15 mg.	Warfarin: 8064	Embolic stroke, and arterial embolism (event/100 person‐years)	Rivaroxaban: 11.2/100 patient‐years Dabigatran: 10.6/100 patient‐years Warfarin: 6.2/100 patient‐years	Not reported
Rivaroxaban	Coleman et al.^52^ 2019	Retrospective cohort	1.4	Rivaroxaban: 1896	ESRD on HD or CKD stage 4–5 (88% CKD 5 or HD)	38.7% received <20 mg/d	Warfarin: 4848	Ischemic stroke/hemorrhagic stroke	Rivaroxaban: 0.85/100 patient‐yearsWarfarin: 1.44/100 patient‐years	0.67 (0.30–1.50)
Rivaroxaban	Lin et al.^53^ 2021	Retrospective cohort	Rivaroxaban:1.6 Warfarin: 2.3	Rivaroxaban: 173 10 mg: 88 (50.8%) 15 mg: 67 (38.7%) 20 mg: 18 (10.4%)	ESRD (82.5% dialysis patients)	Rivaroxaban 10 mg/15 mg/20 mg	Warfarin: 3185	Ischemic stroke or systemic embolism (composite)	Rivaroxaban: 3.6/100 patient‐years Warfarin: 7.1/100 patient‐years Rivaroxaban: 10 mg: 5.7/100 patient‐years	Rivaroxaban versus warfarin: 0.38 (0.17–0.82) Rivaroxaban 10 mg versus warfarin: 0.33 (0.13–0.89)
Rivaroxaban	De Vriese et al.^54^, ^55^ 2020 + 2021 (Valkyrie study)	RCT (open‐label)	1.5 Median follow‐up (extended enrollment) (interquartile range): 1.88 (1.01–3.38)	Rivaroxaban: 46 Rivaroxaban + vitamin K supplements: 42		3 treatments 1) Warfarin (INR 2–3) 2) Daily 10 mg rivaroxaban 3) Daily 10 mg rivaroxaban + vitamin K2 2000 *𝜇g*. Intake 3x weekly during 18 months	Warfarin: 44	Composite of fatal cardiovascular disease and non‐fatal stroke, cardiac events, and other vascular events Ischemic stroke, hemorrhagic stroke	Warfarin: 7.6/100 patient‐years (stroke 1.5 years) Rivaroxaban: 2.3/100 patient‐years (stroke 1.5 years) Warfarin: 63.8/100 patient‐years (composite) Rivaroxaban: 26.2/100 patient‐years (composite) Rivaroxaban + vitamin K2: 21.4/100 patient‐years (composite)	Stroke (1.5 years): Rivaroxaban versus warfarin: No difference Composite extended phase: Rivaroxaban versus warfarin: 0.41 (0.25–0.68) Composite extended phase: Rivaroxaban + vitamin K2 versus warfarin: 0.34 (0.19–0.61)

Abbreviations: BID, (bis in die) = twice a day; ESRD, end‐stage renal disease; HD, hemodialysis; NOAC, non‐vitamin K oral anticoagulant, PD, peritoneal dialysis; RCT, randomized control trial.

**TABLE 3:** Major bleeding outcomes for NOAC use in dialysis patients and characteristics of 11 studies included in the review

NOAC	Study and year of publication	Study design	Mean follow‐up (years)	Treatment group (number of patients)	Renal function	Oral dose	Comparison group (number of patients)	Definition of major bleeding	Incidence: Treatment group and comparison group	HR of treatment group versus comparison group (95% cl)
Apixaban	Pokorney et al.^47^ 2019 (RENAL‐AF)	RCT	1	Apixaban: 82 5 mg BID: 55 2.5 mg BID: 22 Apixaban reduced from 5.0–2.5 mg BID 15	ESRD on HD	5 mg or 2.5 mg BID	Warfarin: 72	ISTH criteria^a^	Apixaban: 8.5% Warfarin: 9.7%	Not reported^b^
Apixaban	Stanton et al.^48^ 2017	Retrospective cohort	Apixaban: 1.01 Warfarin: 1.54	Apixaban: 73 5 mg BID or 10 mg BID: 27 2.5 mg BID: 45	HD/PD/CrCL<25 mL/min or SCr of >2.5 mg	10 mg or 5 mg or 2.5 mg BID	Warfarin: 73	ISTH criteria^a^	Apixaban: 9.6% Warfarin: 17.8%	No difference
Apixaban	Sarrat et al.^56^ 2017	Retrospective cohort	Not reported Approx. 1–4	Apixaban: 40 5 mg BID: 17 2.5 mg BID: 23	ESRD on HD	5 mg or 2.5 mg BID	Warfarin 120	ISTH criteria^a^	Apixaban: 0% Warfarin: 5.8%	Not reported^b^
Apixaban	Reed et al.^49^ 2018	Retrospective cohort	0.83	Apixaban: 74 5 mg BID: 59 2.5 mg BID: 15	ESRD on HD/PD	5 mg or 2.5 mg BID	Warfarin: 50	ISTH criteria^a^	Apixaban: 5.4% Warfarin 22.0%	0.15 (0.05–046)^c^
Apixaban	Siontis et al.^34^ 2018	Retrospective cohort	Apixaban: 0.29 Warfarin: 0.43	Apixaban: 2351 5 mg BID: 1034 2.5 mg BID: 1317	ESRD on HD or PD	5 mg or 2.5 mg BID	Warfarin: 7053	ISTH criteria^a^	Apixaban versus warfarin: Apixaban 19.7/100 patient‐years Warfarin 22.9/100 patient‐years Apixaban 5 mg BID versus warfarin: Apixaban 18.3/100 patient‐years Warfarin 21.9/100 patient‐years Apixaban 2.5 mg BID versus warfarin: Apixaban 20.8/100 patient‐years Warfarin 24.6/100 patient‐years	Apixaban versus warfarin: 0.72 (0.59–0.87) Apixaban 5 mg BID versus warfarin: 0.71 (0.53–0.95) Apixaban 2.5 mg BID versus warfarin: 0.71 (0.56–0.91) Apixaban: 5 mg BID versus 2.5 mg BID: 0.98 (0.68–1.42)
Apixaban	Mavrakanas et al.^50^ 2020	Retrospective cohort	0.31	Apixaban: 521 5 mg BID: 207 2.5 mg BID: 257	PD or HD	5 mg or 2.5 mg BID	No anticoagulation: 1561	Any bleeding resulting in death; any bleeding occurring at a critical site (intracranial, intraocular, retroperitoneal, intra‐articular, pericardial, airway); or any gastrointestinal, urinary tract, or gynecologic bleeding requiring hospitalization.	Apixaban versus non‐anticoagulant treatment: Apixaban 4.9/100 patient‐years Warfarin 1.6/100 patient‐years Apixaban 5 mg versus no treatment: Apixaban 5 mg 9.8/100 patient‐years No anticoagulation users: 1.7/100patient‐years Apixaban 2.5 mg versus no treatment: Apixaban 2.5 mg 2.9/100 patient‐years No anticoagulation users 1.4/100 patient‐years	Apixaban versus non‐anticoagulant treatment: 2.74 (1.37–5.47) Apixaban 5 mg versus no treatment: 4.61 (1.91–11.15) Apixaban 2.5 mg versus no treatment: 2.02 (0.58–7.04)
Rivaroxaban and apixaban	Miao et al.^51^ 2020	Retrospective cohort	0.87	Rivaroxaban: 787 Apixaban: 1836	ESRD on HD	Rivaroxaban: < 20 mg/day Apixaban: <10 mg/day			Rivaroxaban: 3.73/100 patient‐years Apixaban: 3.49/100 patient‐years	1.00 (0.63–1.58)
Rivaroxaban and dabigatran	Chan et al.^9^ 2015	Retrospective cohort	Dabigatran:0.44 Rivaroxaban: 0.30Warfarin: 0.48	Rivaroxaban: 244 Dabigatran: 281	ESRD on HD	20 mg/15 mg.	Warfarin: 8064	Hemorrhagic event resulting in hospitalization or death.	Rivaroxaban: 68.4/100 patient‐years. Bleeding mortality rate 16.2 deaths/100 patient‐years. Dabigatran: 83.1/100 patient‐years. Bleeding mortality rate 19.2 deaths/100 patient‐years. Warfarin: 47.1 events/100 patients‐years. Bleeding mortality rate 10.2 deaths/100 patient‐years.	Not reported^b^
Rivaroxaban	Coleman et al.^52^ 2019	Retrospective cohort	1.4	Rivaroxaban: 1896	CKD stage 4–5 and HD (88% CKD 5 or HD)	38.7% received <20 mg/d	Warfarin: 4848	Bleeding related hospitalization.	Rivaroxaban: 3.73/100 patient‐years Warfarin: 6.16/100 patient‐years	32% (1–53%)
Rivaroxaban	Lin et al.^53^ 2021	Retrospective cohort	Rivaroxaban: 1.6 Warfarin: 2.3	Rivaroxaban: 173 10 mg: 88 (50.8%) 15 mg: 67 (38.7%) 20 mg: 18 (10.4%)	ESRD (82.5% dialysis patients)	Rivaroxaban 10 mg/15 mg/20 mg	Warfarin: 3185	Bleeding related hospitalization based on ISTH criteria	Rivaroxaban: 8.4/100 patient‐years Warfarin: 7.7/100 patient‐years Rivaroxaban 10 mg: 5.7/100 patient‐years	Rivaroxaban versus warfarin: 0.88 (0.51–1.51) Rivaroxaban 10 mg versus warfarin:0.60 (0.25–1.44)
Rivaroxaban	De Vriese et al.^54^, ^55^ 2020 + 2021 (Valkyrie study)	RCT (Open‐label)	1.5 Median follow‐up (extended enrollment) (interquartile range): 1.88 (1.01–3.38)	Rivaroxaban: 46 Rivaroxaban + vitamin K supplements: 42	HD	3 treatments 1) VKA target INR 2–3. 2)Daily 10 mg rivaroxaban 3)Daily 10 mg rivaroxaban + vitamin K2 2000 𝜇g. Intake 3x weekly for 18 months	Warfarin: 44	Life‐threatening bleeding: Fatal bleeding, symptomatic intracranial bleeding, a decrease in hemoglobin of 5 g/dL or more, or a requirement for transfusion of ≥4 units of blood, inotropic agents, or surgery. Major bleeding: A requirement for transfusion of ≥2 units of blood or a decrease in hemoglobin of ≥2 g/dL, and not fulfilling the criteria for life‐threatening bleeding. All other bleedings were regarded as minor.	Bleeding (Life‐threatening+major) 1.5 years: Warfarin: 36/100 patient‐years Rivaroxaban: 15.4/100 patient‐years Extended follow‐up: Rivaroxaban: 13.8/100 patient‐years VKA: 22.7/100 patient‐years Rivaroxaban+Vitamin K2: 3.8/100 person‐years	Extended follow‐up: Rivaroxaban (pooled group) versus warfarin: 0.44 (0.23–0.85) Rivaroxaban versus warfarin: 0.39 (0.17–0.90) Rivaroxaban + vitamin K2 versus warfarin: 0.48 (0.22–1.08)

Abbreviations: BID, (bis in die) = twice a day; CKD, chronic kidney disease; ESRD, end‐stage renal disease; HD, hemodialysis; NOAC, non‐vitamin K oral anticoagulant, PD, peritoneal dialysis; RCT, randomized control trial, VKA, vitamin K antagonist.

^a^ ISTH criteria: composite of any acute, clinically overt bleeding plus at least one of the following: a decrease in hemoglobin of 2 g/dl or more, bleeding that required the transfusion of 2 units or more of blood products (packed red blood cells or fresh frozen plasma), bleeding in at least one critical site (intracranial, intraspinal, intraocular, pericardial, intra‐articular, intramuscular with compartment syndrome, or retroperitoneal), or fatal bleeding.

^b^ Authors did not report HR.

^c^ Adjusted odds ratio.

**TABLE 4:** Overview of upcoming trials in dialysis patients

Trial name	ClinicalTrials.Gov identifier	Country of origin	Number (patients)	Study design	Intervention	Comparator	Primary endpoints	Expected completion month/year
AXADIA	NCT02933697	Germany	108 (HD)	RCT (Open‐label)	Apixaban 2.5 mg BID	VKA (Phenprocoumon)	Thromboembolic events Major, non‐major, and specific bleedings (e.g., after shunt removal)	July 2023
SAFE‐D	NCT03987711	Canada	150 (HD/PD)	RCT (Open‐label)	Apixaban 2.5 mg BID And 5.0 mg BID	VKA (Warfarin) and No anticoagulants	Stroke and systemic embolism Major bleeding	December 2021
DANWARD	NCT03862859	Denmark	718 (HD/PD)	RCT (Open‐label)	VKA (Warfarin)	No treatment	Transient ischemic attack, fatal, and non‐fatal ischemic or unspecific stroke. Death to either ischemic or unspecified stroke Major bleeding	September 2024
ApiDP	NCT04006093	France	24 (PD/normal renal function)	Open‐label Non‐randomized pharmacokinetic study	Apixaban 5.0 mg BID	Participants with normal renal function.	Apixaban areal under the curve Apixaban maximum plasma concentration (Measurement of apixaban plasma concentration at different times)	February 2020 (Unpublished results)
AVKDIAL	NCT02886962	France	855(HD)	RCT (Open‐label)	VKA	No treatment	Thrombosis and severe bleeding	January 2023
SACK	Not available	Sweden	1,500 (eGFR<15 and dialysis)	RCT (Open‐label)	Apixaban 2.5 mg BID	No treatment	Ischemic stroke and systemic thrombosis Bleeding (intracranial and fatal bleeding	2026

Abbreviations: BID, (bis in die) = twice a day; HD, hemodialysis; PD, peritoneal dialysis; RCT, randomized control trial, VKA, vitamin K antagonist.

### 3.2 Quality assessment

Assessment of the quality of all cohort studies using this rank system resulted in nine high‐quality studies with all studies receiving between seven and nine stars. Cochrane ROB tool resulted in one RCT with low risk of bias and one RCT with high risk of bias (See supporting information).

### 3.3 Apixaban PK studies in dialysis patients

Four studies investigated apixaban PK in HD patients.^38^, ^39^, ^40^, ^41^ Wang et al.^38^ showed that administration of single dose of 5 mg apixaban immediately after a HD session increased the area under the curve (AUC) with 36% in HD patients compared with non‐renal patients. Maximum concentration level predialysis and postdialysis only increased negligibly perhaps due to clearance of apixaban via the dialysate (Table 1). Mavrakanas et al.^39^ also studied PK of apixaban in HD patients and showed that multiple doses (2.5 mg twice daily) caused a higher accumulated apixaban concentration in steady state compared with the single first day dose. A dose of 2.5 mg twice daily in HD patients was similar to 5 mg apixaban twice daily in healthy subjects. Furthermore, they found that 5 mg apixaban led to supratherapeutic levels in HD patients (Table 1). Van den Bosch et al.^40^ compared PKs in HD patients exposed to 2.5 or 5 mg apixaban twice daily comparing predialysis and postdialysis dosing. Administration of apixaban 30 min before a HD session gave lower exposure compared with postdialysis administration. Notably, Van den Bosch et al. administered the dose 30 min predialysis, whereas Wang et al.^38^ gave apixaban 2 h before a dialysis session. Wang et al.^38^ observed 14% reduction in AUC when comparing pre‐HD and post‐HD concentration, while Van den Bosch et al.^40^ observed a 48% reduction in AUC (Table 1). Pokorney et al.^41^ evaluated in a substudy to the RENAL‐AF trial the PK of apixaban collected over 1 month in 64 HD patients with AF treated with 5 mg apixaban twice daily or 2.5 mg apixaban twice daily if patients were ≥80 years or weighed ≤60 kg. Steady‐state exposure was modestly higher in HD patients; however, the results were quite similar to apixaban levels in non‐ESRD patients from the ARISTOTLE^14^ trial (Table 1).

### 3.4 Rivaroxaban PK studies in dialysis patients

Two studies investigated PK of rivaroxaban in HD patients.^42^, ^43^ Dias et al.^43^ found that a single dose of 15 mg rivaroxaban daily increased AUC 56% 3 h postdialysis. Notably, PK was not influenced by HD (only 5% lower AUC when comparing predialysis with postdialysis AUC) as shown in Table 1. De Vriese et al.^42^ investigated PKs of single and multiple doses of rivaroxaban in HD patients. AUC level in HD patients after a 10 mg single dose was similar to a 20 mg single dose in healthy subjects. Moreover, rivaroxaban was not removed by dialysis, and no accumulation occurred after multiple doses of 10 mg in HD patients (Table 1).

### 3.5 Dabigatran PK studies in dialysis patients

Two studies investigated PK of dabigatran in HD patients.^44^, ^45^ Both studies showed that dabigatran was removed during HD. Khadzhynov et al.^44^ showed that a minor redistribution of dabigatran occurred in the plasma after dialysis had ended. Stangier et al.^45^ found twofold accumulation of dabigatran in HD subjects, and moreover, the drug was sixfold accumulated in subjects with severe renal impairment compared with healthy subjects (Table 1).

### 3.6 Edoxaban PK studies in dialysis patients

Only one study was found. Parasrampuria et al.^46^ investigated edoxaban PK in an open‐label phase 1, randomized crossover study in HD patients. The patients received a single dose of edoxaban 15 mg 2 h prior to HD or in between HD sessions (off‐dialysis). HD caused a minor decrease in AUC but did not affect edoxaban levels significantly. HD was deemed ineffective in terms of edoxaban removal (Table 1). Different doses or prolonged treatment was not tested.

### 3.7 Apixaban stroke and bleeding outcomes in dialysis patients

The only apixaban RCT, RENAL‐AF,^47^ showed that apixaban 2.5 or 5 mg twice daily resulted in similar rates of stroke and bleeding as warfarin‐treated HD patients (Tables 2 and 3). Unfortunately, the study was stopped earlier than planned. Only 154 patients were enrolled (out of the 760 patients originally planned) causing it to be significantly underpowered and difficult to interpret. Four^34^, ^48^, ^49^, ^56^ retrospective cohort studies compared apixaban with warfarin in ESRD patients on dialysis. Only three^34^, ^48^, ^56^ of them included PD patients. In terms of stroke prevention, Stanton et al.^48^ and Reed et al.^49^ compared apixaban with warfarin treatment and found similar stroke occurrence (Table 2). The largest cohort study by Siontis et al.^34^ also found similar stroke rates in matched cohorts of dialysis patients with AF treated with either apixaban or warfarin. A lower risk of stroke was found with 5 mg twice daily compared with either 2.5 mg twice daily apixaban or warfarin (Table 2). Mavrakanas et al.^50^ compared apixaban treatment (both 5 mg and 2.5 mg twice daily) with no anticoagulation treatment in dialysis patients with AF. Interestingly, apixaban use was not associated with a lower risk of stroke compared with no anticoagulant therapy. A trend toward fewer ischemic strokes was seen with apixaban, but it was offset by a trend toward more hemorrhagic strokes especially with 5 mg twice daily. Thus, compared with patients who did not receive any anticoagulants, a significantly higher incidence of fatal or intracranial (major) bleeding was seen in the subgroup of patients treated with standard apixaban dose (5 mg twice daily) but not in patients who received the reduced apixaban dose (2.5 mg twice daily) (Tables 2 and 3). Despite lower risk of bleeding, low dose was also associated with an increased risk of ischemic stroke or myocardial infarction compared with no anticoagulation. Stanton et al.^48^ and Sarratt et al.^56^ found that apixaban was associated with a similar risk of major bleeding as warfarin treatment (Table 3). Siontis et al.^34^ and Reed et al.^49^ found lower risk of major bleeding when apixaban was compared with warfarin. Siontis et al.^34^ also reported that both 5 and 2.5 mg twice daily were associated with significantly lower risk of major bleeding compared with warfarin (Table 3). Finally, a retrospective cohort study by Miao et al.^51^ compared apixaban with rivaroxaban in HD patients and found no significant difference regarding the risk of major bleeding and stroke but did not compare the outcomes with warfarin‐treated patients (Tables 2 and 3).

### 3.8 Rivaroxaban stroke and bleeding outcomes in dialysis patients

The Valkyrie study^54^, ^55^ was the only RCT found that compared rivaroxaban with warfarin and assessed bleeding, stroke, and calcification outcomes in HD patients. Progression of vascular calcification was similar in rivaroxaban, rivaroxaban plus vitamin K supplement, and warfarin‐treated HD patients. Although not powered for events, the risk of major bleeding was lower with rivaroxaban compared with warfarin‐treated patients (Table 3). The occurrence of stroke was reported to be similar in both rivaroxaban and warfarin‐treated patients, and in the extended follow‐up, rivaroxaban significantly reduced the composite outcome of fatal and non‐fatal cardiovascular events and major bleeding complications compared with VKA (Table 3). Three^9^, ^52^, ^53^ retrospective observational studies compared rivaroxaban and warfarin. For HD and CKD 4–5 patients, Coleman et al.^52^ showed that stroke prevention on rivaroxaban was similar to warfarin treatment but the risk of major bleeding was reduced 32% in the rivaroxaban‐treated compared with warfarin‐treated patients. Lin et al.^53^ found lower risk of stroke or systemic embolism in rivaroxaban‐treated ESRD patients (82.5% dialysis patients) compared with warfarin‐treated and similar incidence of major bleeding. Contrary to this, Chan et al.^9^ showed that the event rate of stroke and major bleeding was higher in rivaroxaban‐treated compared with warfarin‐treated HD patients (Tables 2 and 3).

### 3.9 Dabigatran stroke and bleeding outcomes in dialysis patients

Only one retrospective cohort study by Chan et al.^9^ compared dabigatran and warfarin treatment in HD patients. Dabigatran‐treated patients had a significantly higher bleeding risk including fatal events compared with warfarin‐treated patients. The stroke rate was similar; however, according to the authors, there were too few strokes in the cohort to detect meaningful differences in stroke and arterial embolism between these two groups. (Tables 2 and 3).

### 3.10 Upcoming studies in dialysis patients

Table 4 shows upcoming RCTs investigating NOACs and VKA in HD and PD patients, and one finished unpublished PK study in PD patients. All upcoming NOAC‐trials use apixaban.

## 4 DISCUSSION

This study reviewed the existing literature on the use of NOACs in dialysis patients. Of the 20 studies included, nine were PK studies. Only two studies were RCTs, and the majority (nine) were retrospective cohort studies. Six studies were recruiting/ongoing (five RCTs) or without results (one PK study). PK studies in dialysis patients showed that multiple doses of apixaban (2.5 mg twice daily) gave higher exposure at steady state compared with single dose. The RENAL‐AF PK substudy^41^ showed that 5 mg apixaban twice daily or 2.5 mg apixaban twice daily if patients were ≥80 years or weighed ≤60 kg was similar to apixaban levels in non‐ESRD patients.^14^, ^41^ However, the results were only extracted from an abstract with limited description of data collection and methodology. More valid data on apixaban PK can be found in the PK study by Mavrakanas et al.^39^ despite a lower number of patients. This study showed that treatment for 8 days resulted in significant accumulation with both 2.5 and 5 mg apixaban twice daily. Especially 5 mg twice daily led to supratherapeutic levels, whereas the reduced dose of 2.5 mg twice daily resulted in drug exposure that was comparable with that of the standard dose in patients with preserved renal function. Interindividual variability could be underestimated due to the low number of patients, but unlike most other PK studies, multiple doses were given over 8 days which is arguably better compared with single dose studies when assessing drug accumulation. Thus, the best apixaban PK study suggests that a dose of 5 mg twice daily should be avoided in dialysis patients regardless of age and weight.

Considering the PK of rivaroxaban, the use of 10 mg in ESRD dialysis patients resulted in plasma concentration similar to 20 mg rivaroxaban in healthy subjects from the ROCKET‐AF study. No accumulation was seen after multiple doses of 10 mg daily.^42^ Dabigatran was removed by dialysis; however, minor redistribution was seen after HD was ended, which indicates accumulation of dabigatran which therefore should be avoided in ESRD patients.^44^ Edoxaban is not removed by dialysis but whether accumulation occurs over time with prolonged treatment has not been tested.^46^ Further PK studies are needed in order to establish Edoxaban dosage in ESRD, which could guide adequately sized RCTs in which stroke and bleeding outcomes could be assessed.

RENAL‐AF is so far the largest NOAC RCT in dialysis patients and investigated apixaban versus warfarin treatment in HD patients with AF. Unfortunately, it was prematurely stopped due to funding problems and results have not been subjected to peer review in a journal.^47^ Originally, the study planned to enroll 760 HD patients but only accomplished enrollment of 154 patients (20%). Thus, although indisputably underpowered, it showed that 5 mg apixaban twice daily had similar risk of stroke and bleeding as warfarin. It should be mentioned that time in therapeutic international normalized ratio (INR) range in the warfarin group was only 44% with a significant proportion of patients in the subtherapeutic range. Unfortunately, it failed to demonstrate whether 2.5 mg apixaban twice daily lowered the bleeding risk with similar stroke prevention as warfarin treatment or whether 2.5 mg was better or equal to 5 mg twice daily. Notably, 15 patients had apixaban dose reduced from 5 mg twice daily to 2.5 mg twice daily after randomization.^41^, ^47^ Apixaban dose was already reduced to 2.5 mg if patients were aged ≥80 years or weighed ≤60 kg. Accordingly, like in non‐dialysis patients, it is relevant to consider both age and weight of the patient when using apixaban to minimize the risk of bleeding, but as mentioned previously, the PK studies suggest that 5 mg twice daily should be avoided due to accumulation. The fact that 27% of patients in the apixaban arm were reduced from 5 to 2.5 mg twice daily seems to substantiate this.

Siontis et al.^34^ did the largest cohort study. More than 25,000 dialysis patients with AF were included for comparison of apixaban (*n =* 2,351) with warfarin (*n =* 23,172). The study reported that apixaban was associated with similar risk of stroke but less risk of bleeding compared with warfarin. The study was also able to differentiate stroke and bleeding outcomes based on apixaban dose. Both 5 and 2.5 mg twice daily were associated with significantly lower risk of major bleeding compared with warfarin, whereas stroke prevention was best with 5 mg twice daily in comparison with either reduced dose (2.5 mg twice daily) or warfarin. A large and broad study population is a big advantage regarding bleeding and stroke outcomes compared with the smaller studies.^48^, ^49^, ^56^ By sheer size, the findings by Siontis et al.^34^ have more weight but several limitations should be mentioned. Due to the observational design, residual selection bias likely affected the results to some extent. Apixaban adherence and quality of the VKA treatment (time in therapeutic range) were not included. Discontinuation rates were high (about two thirds of patients in each group were no longer taking the anticoagulant 1 year after the initial prescription), and the observation period was short (mean follow‐up <1 year). Collectively, these aspects make the comparison between apixaban and VKA difficult and highlights the need of adequately sized RCTs.

Comparison of NOAC (apixaban) treatment versus no anticoagulant therapy in dialysis patients with AF was investigated by Mavrakanas et al.^50^ In this study, apixaban did not lower the risk of stroke compared with non‐treated regardless of apixaban dosage and could reflect poor treatment adherence. The risk of fatal or intracranial bleeding events was higher on apixaban compared with no treatment especially with 5 mg apixaban twice daily. Ideally, VKA treated should have been included as a third group to facilitate comparison with the study by Siontis et al.^34^ Overall, comparison with the latter study is difficult due to a markedly lower stroke incidence most likely caused by methodological differences such as patient population (incident vs. prevalent population) and outcome definition (ischemic stroke vs. any stroke). Yet, the fact that fewer bleeding events were observed with the reduced dose compared with the standard dose, in line with PK data, suggest that the low dose regimen of apixaban potentially could be a better treatment option compared with standard dose and VKA treatment.

Interestingly, Europe and the United States treat dialysis patients differently in terms of apixaban. FDA has approved use of apixaban in dialysis patients,^33^ and a growing number of patients are now treated with apixaban which in 2015 accounted for roughly 25% of new anticoagulation prescriptions for ESRD patients in the United States.^34^ The FDA approval has been met with some skepticism due to a lack of apixaban RCTs in ESRD. FDA approval was allegedly primarily based on PK data originating from a small study by Wang et al.^38^ in which eight HD patients and eight healthy subjects were treated with apixaban 5 mg resulting in comparable maximum blood concentrations and anti‐factor Xa activity.^34^, ^38^ So far, the European Medicines Agency (EMA)^57^ has not approved apixaban for use in patients with eGFR<15 ml/min including dialysis patients.

Three cohort studies compared rivaroxaban with warfarin with somewhat different results.^9^, ^52^, ^53^ The largest of these by Coleman et al. found similar stroke prevention and lower risk of bleeding with rivaroxaban compared with warfarin in a mixed cohort primarily consisting of CKD 5 and dialysis patients.^52^ Contrary to this, in a cohort of HD patients, Chan et al.^9^ found that rivaroxaban was associated with increased risk of both stroke and bleeding compared with warfarin treatment. The Valkyrie study^54^ was the only RCT investigating rivaroxaban. Despite of the relatively small sample size, the study was a well‐designed RCT with a long follow‐up period of 18 months plus an extended follow‐up phase.^55^ Moreover, the choice of 10 mg rivaroxaban dose was based on a dose‐finding study, although one might argue that stroke prevention potentially could be suboptimal with a 10 mg dose. The two cohort studies mentioned above both reported higher rivaroxaban dose.^9^, ^52^ In contrast, the Taiwanese cohort study^53^ found a lower risk of stroke or systemic embolism alongside a similar incidence of bleeding as warfarin treated in the subgroup of patients treated with 10 mg of rivaroxaban. The incidence of major bleeding and stroke outcomes may be more evident in a larger study with more patients, yet the results from the Valkyrie study seem promising. Thus, in dialysis patients, low dose rivaroxaban (10 mg) may be a suitable alternative to VKA, but larger rivaroxaban RCTs are required prior to widespread use.

When assessing the bleeding risk in dialysis patients treated with VKA or NOAC, it is important to consider whether VKA or NOACs were given together with other drugs affecting hemostasis. Thus, due to the high prevalence of antiplatelets and intermittent use of LMWH in HD patients,^58^ results may represent the effects of a cocktail of drugs rather than just NOAC or VKA. Accordingly, bias due to concomitant antiplatelet therapy applies to most cohort studies although some tried to adjust for this. On the other hand, the large cohort studies provide us with real‐life data from a broad spectrum of dialysis patients, compared with the cherry‐picked patients that are eligible for RCTs and thus tend to be more fit with less comorbidity and smaller pill burden including antiplatelets. Another important aspect when comparing NOACs with VKA is the quality of the VKA treatment in terms of the time within the therapeutic range (typically INR 2–3).^59^ Thus, if time in therapeutic range is low, there is lack of anticoagulation and thereby a higher risk of stroke, whereas INR above 3.5 increases the risk of bleeding.^21^, ^60^ Provided that time in therapeutic range is low on VKA treatment, NOAC may inevitably be a better alternative both when assessing stroke prevention and the risk of bleeding. Retrospective cohort studies are usually unable to account for this aspect unlike RCTs. The overall low life expectancy in the dialysis population should also be considered. Patients may die before they harvest the benefit of stroke prevention due to anticoagulants but with a substantial augmented risk of major bleeding events.

The criteria used for starting anticoagulant therapy regardless of NOAC or VKA are debatable and vary between studies. In terms of AF in the dialysis population, the risk of stroke due to short lasting episodes of asymptomatic AF (e.g., detected by continuous monitoring) might be substantially lower than that of permanent AF or symptomatic AF diagnosed by usual care yet with a similar risk of bleeding if anticoagulant therapy is initiated. Furthermore, anticoagulant treatment does not eliminate the risk of stroke.^50^, ^61^ In some patients, especially those prone to bleeding, alternatives such as left atrial appendage occlusion could be a better alternative instead of anticoagulant therapy.^62^, ^63^

Currently, three upcoming RCTs will investigate use of apixaban in ESRD patients. ALAXIA compares apixaban versus phenprocoumon in HD patients, whereas SAFE‐D compares apixaban with warfarin or no anticoagulants in both HD and PD patients. The Swedish SACK trial will compare apixaban versus no anticoagulation in patients with eGFR <15 +/− dialysis.^35^ In addition, the AVKDIAL and DANWARD trials will investigate VKA versus no anticoagulant treatment in dialysis patients with AF. Until then, available evidence suggests a potential for use of apixaban and rivaroxaban in reduced dose as an alternative to VKA in dialysis patients if anticoagulant treatment is deemed necessary.

### 4.1 Limitations

The biggest limitation of this review is the lack of adequately sized RCTs. Only two RCTs were found, and although RCTs are considered superior in design to retrospective observational cohort studies, both RCTs enrolled a relatively small number of patients. All PK studies had strict inclusion and exclusion criteria typically excluding, for example, patients with liver disease, gastrointestinal disease, or cardiovascular disease. This obviously limits generalizability to the real‐world dialysis population in terms of NOAC treatment. In contrast to the relatively small number of patients in the PK studies, the retrospective cohort studies had a higher number of patients thereby representing a much broader spectrum of the dialysis population; however, due to the observational nature, causality is not always straightforward and selection bias is hard to avoid. Adherence rates among NOAC treated and time in therapeutic range in VKA treated are usually not reported in cohort studies and are obvious limitations. In addition, cohort studies could be biased by concomitant antiplatelet therapy, lack of accurate medical reports, misclassification, and confounding by indication. Relatively, few studies enrolled PD patients, only three cohort studies included PD patients, and only one PK study in PD patients was identified but without published results so far. Thus, the potential for drug removal via PD dialysis fluid remains unclear, which could be relevant to investigate in upcoming studies. So far, it is unclear whether NOAC safety outcomes differ in PD patients. Finally, it could be perceived as a limitation that CKD stage 4–5 patients were excluded in this review. Especially, RCTs conducted in patients with CKD stage 4–5 are relevant when studying the safety outcomes of NOACs since these patients usually begin dialysis treatment if not transplanted. Currently, there are no RCTs in CKD stage 4–5 patients applicable to the dialysis population.

## 5 CONCLUSION

This systematic review showed that of all four NOACs apixaban and rivaroxaban in low dose appear to be the best candidates for safe use in dialysis patients. Firstly, the PK properties of NOACs vary and apixaban and rivaroxaban were associated with less drug accumulation compared with edoxaban and dabigatran. Secondly, apixaban and rivaroxaban were evaluated in the only two RCTs conducted so far with similar stroke rates as warfarin treated and without significantly more bleeding events. Yet, caution is advised since both studies were underpowered regarding stroke and bleeding outcomes. Cohort studies comparing apixaban high dose (5 mg) with low dose (2.5 mg) suggest a lower risk of stroke with high dose but also a higher risk of bleeding with high dose apixaban. Apixaban versus no anticoagulation was compared in one cohort study and did not lower the risk of stroke compared with non‐treated regardless of apixaban dosage. Cohort studies comparing rivaroxaban with VKA yielded conflicting results. Currently, widespread use of NOACs in dialysis patients is limited by adequately sized RCTs, but several apixaban trials are underway. Clinicians are advised to individualize treatment and carefully weigh the risk of stroke versus bleeding especially in patients with a prior history of bleeding before prescribing NOACs to dialysis patients.

## Supporting information


**Figure S1.** Overview of different search‐terms used in the systematic literature search.
**Table S1.** Detailed Newcastle Ottawa Scale of each included cohort study.
**Table S2.** Detailed Cochrane risk of bias tool for included randomized controlled trials.
