# Supplementary material for: The use of non‐vitamin K oral anticoagulants in dialysis patients—A systematic review
Source: Semin Dial. 2022 May 27;35(6):463–80. doi: 10.1111/sdi.13098 (PMC9796794; doi:10.1111/sdi.13098)
Supplement: Supplementary file 1 — Figure S1. Overview of different search‐terms used in the systematic literature search. Table S1. Detailed Newcastle Ottawa Scale of each included cohort study. Table S2. Detailed Cochrane risk of bias tool for included randomized controlled trials. [file SDI-35-463-s001.docx]

# **Supplements**

List of abbreviations

| **AF** | Atrial fibrillation |
| --- | --- |
| **AHA** | American Heart Association |
| **ARISTOTLE** | Apixaban for Reduction in Stroke and Other Thromboembolic Events in Atrial Fibrillation |
| **AUC** | Area under the curve |
| **BID** | Bis in die (twice a day) |
| **CKD** | Chronic kidney disease |
| **ClCr** | Creatinine clearance |
| **C_max_** | Maximum plasma concentration |
| **ENAGE-AF-TIMI 48** | Effective Anti-coagulation with Factor Xa Next Generation In Atrial Fibrillation-Trombolysis In Myocardial Infarction 48 |
| **ESRD** | End stage renal disease |
| **DOAC** | Direct oral anticoagulant |
| **HD** | Hemodialysis |
| **INR** | International normalized ratio |
| **LMWH** | Low-molecular-weight heparin |
| **NOAC** | Non-vitamin K antagonist/novel oral anticoagulant |
| **RE-LY** | Randomized Evaluation of Long-term Anticoagulation Therapy |
| **ROCKET AF** | Rivaroxaban Once-daily, Oral, Direct Factor Xa inhibition Compared with vitamin K Antagonism for prevention of Stroke and Embolism Trials in Atrial Fibrillation |
| **PD** | Peritoneal dialysis |
| **PK** | Pharmacokinetic |
| **PRISMA** | Preferred reporting items for systematic reviews and meta-analysis |
| **RCT** | Randomized clinical trials |
| **VKA** | Vitamin K antagonist |

Figure S1

Overview of different search-terms used in the systematic literature search

**
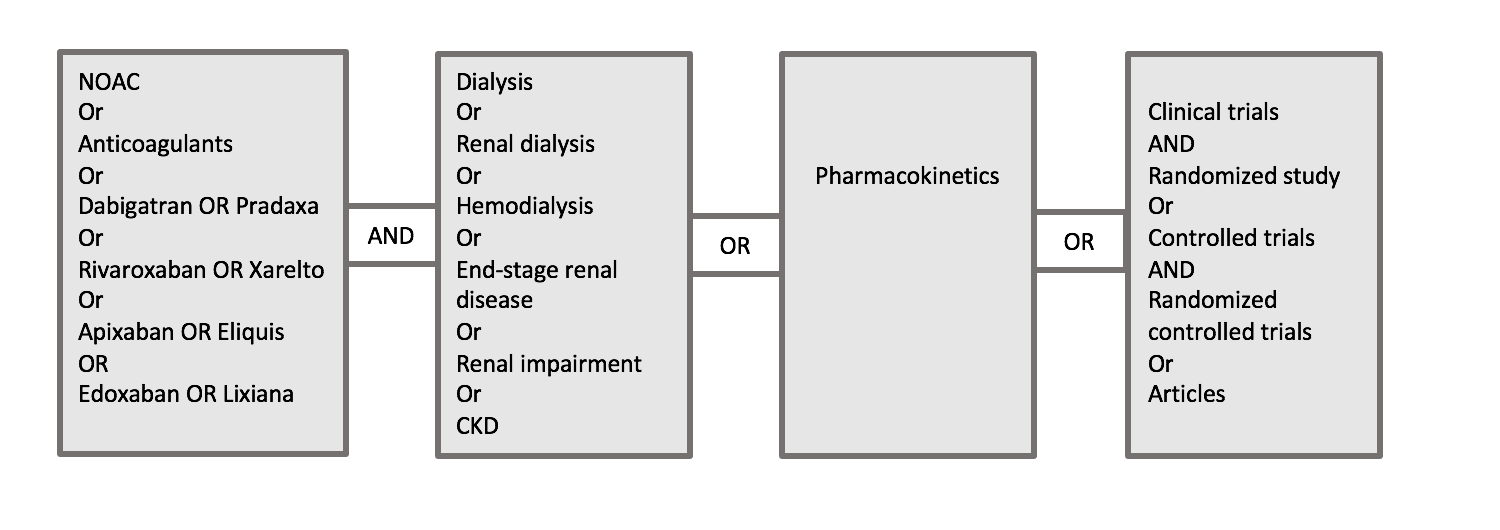
**

The search-terms were usually used with filter functions. Filters like “clinical trials” and “randomized study” were applied in Pubmed, and “controlled trials” and “randomized controlled trials” were applied in Embase. In the Cochrane database, the setting “trials” was selected and in Web of Science database the searching criteria “articles” was applied.

Table S1 **Detailed Newcastle Ottawa Scale of each included cohort study**

|  | **Selection** | | | | | **Comparability** | | | **Outcomes** | | |  |
| --- | --- | --- | --- | --- | --- | --- | --- | --- | --- | --- | --- | --- |
| Study | Representativeness of exposed cohort | Selection of non-exposed cohort | Ascertainment of exposure | Demonstration that outcome of interest was not present at start of study | Main factor | | Additional factors | Assessment of outcome | | Follow-up length | Loss to follow-up rate | Total score |
|  | Representativeness of average HD/PD patients in community (risk of stroke and bleeding) | Drawn from the same community as exposed | Secured records | Stroke or bleeding due to anticoagulant | Study controls for renal function | | Study controls for other factors like age, weight, history of bleeding etc. | Record linkage | |  | Complete follow up (all subjects accounted for) or subjects lost to follow up unlikely to introduce any bias |  |
| Chan et al.^9^  2015 | * | * | * | * | * | | * | * | | * | * | 9 |
| Siontis et al.^31^ 2018 | * | * | * | * | * | | * | * | | * | * | 9 |
| Stanton et al.^45^ 2017 | 0 | * | * | * | * | | * | * | | * | * | 8 |
| Sarrat et al.^46^ 2017 | 0 | * | * | * | * | | * | * | | * | * | 8 |
| Reed et al.^47^ 2018 | 0 | * | * | * | * | | * | * | | * | 0 | 7 |
| Mavrakanas et al.^46^ 2020 | 0 | * | * | * | * | | * | * | | * | 0 | 7 |
| Miao et al.^49^ 2020 | 0 | * | * | * | * | | * | * | | * | 0 | 7 |
| Coleman et al.^52^ 2019 | 0 | * | * | * | * | | * | * | | * | * | 8 |
| Lin et al.^53^  2021 | 0 | * | * | * | * | | * | * | | * | * | 8 |

Table S2 **Detailed Cochrane risk of bias tool for included randomized controlled trials.**

|  | Selection bias | | Reporting bias | Other bias | Performance bias | Detection bias | Attrition bias |
| --- | --- | --- | --- | --- | --- | --- | --- |
|  | Random sequence generation | Allocation concealment | Selective reporting | Other sources of bias | Blinding (participants and personnel) | Blinding (outcome assessment) | Incomplete outcome data |
| Pokorney et al. 2019 (RENAL-AF)^44^ | Unclear risk  (Insufficient information about the process of the randomization sequence) | Unclear risk  (Insufficient information) | Unclear risk  (Insufficient information) | High risk  (Study stopped early, participants loss to follow up, some participants were reduced in dose without any information) | High risk  (Not blinded) | High risk  (Not blinded evaluation of outcomes) | Low risk  (Handling of incomplete outcome data was completed) |
| De Vriese et al. 2020 (Valkyrie study)^50^ | Low risk  (Computer generated randomization schedule) | Low risk  (The investigator who reviewed all CT scans and analyzed the pulse wave analysis curves that were blinded to the treatment allocation) | Low risk  (No selective outcomes are reported) | Low risk | High risk  (Not blinded) | High risk  (Not blinded evaluation of outcomes) | Low risk  (Complete data outcome) |
